# Magnetic resonance imaging for identification of myocardial injury during ablation for atrial fibrillation: first experiences with the Miyabi MRI system

**DOI:** 10.1186/1532-429X-11-S1-O89

**Published:** 2009-01-28

**Authors:** Anil-Martin Sinha, Guido Ritscher, Christian Mahnkopf, Nathan Burgon, Troy J Badger, Martin Schmidt, Harald Marschang, Klaus J Gutleben, Edward VR DiBella, Nassir F Marrouche, Johannes Brachmann

**Affiliations:** 1 Klinikum Coburg, Coburg, Germany; 2 School of Medicine, University Of Utah, Salt Lake City, UT USA; 3 School of Medicine, University of Utah, Salt Lake City, UT USA; 4 Center of Advanced Imaging Research, University of Utah, Salt Lake City, UT USA

**Keywords:** Cardiac Magnet Resonance, Myocardial Injury, Maximum Intensity Projection, Cardiac Magnet Resonance Imaging, Paroxysmal Atrial Fibrillation

## Introduction

Pulmonary vein antrum isolation (PVAI) has become an effective therapy in patients with paroxysmal atrial fibrillation (AF). Extension and location of ablation lesions often remain unclear during the procedure.

## Purpose

We evaluated a new approach on visualization of myocardial injury using cardiac magnet resonance imaging (CMR) during PVAI procedure.

## Methods

Patients who underwent PVAI, received CMR before and at the terminal phase of PVAI using the Miyabi-MRI system (Siemens, Germany). Delayed enhancement (DE-CMR) free breathing sequences were applied, and maximum intensity projections (MIP) obtained. Myocardial injury size was then measured on manually segmented 3D images by a computer algorithm using dynamic thresholding.

## Results

**Figure 1:**
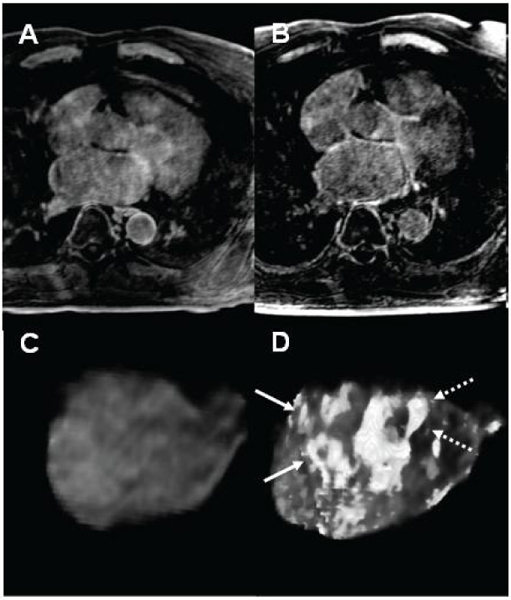
Figure 1

30 patients received PVAI from February to July 2008. In a subset of 14 patients, CMR was performed before and during the procedure. Using DE-CMR, the increase in average lesion to healthy myocardium ratio was 10.3 ± 4.1% during PVAI. Figure 1 shows an example of MIP of a DE-CMR scan in 2D (A, B) and 3D segmentation (C, D) in a anterior view pre (A, C), and during PVAI (B, D). Myocardial injury is identifiable as white tissue around PV single ostia (full arrows) and common trunk (dashed arrows).

## Conclusion

Using CMR is feasible in the course of ablation procedures. In PVAI patients, DE-CMR allowed identification of location and extension of myocardial injury. Therefore, this new CMR approach might improve ablation techniques, and thus long-term success of PVAI.

